# The pros and cons of probiotic use in pediatric oncology patients following treatment for acute lymphoblastic leukemia

**DOI:** 10.3389/fped.2024.1427185

**Published:** 2024-10-22

**Authors:** Miroslava Šimiaková, Viktor Bielik

**Affiliations:** Department of Biological and Medical Science, Faculty of Physical Education and Sports, Comenius University in Bratislava, Bratislava, Slovakia

**Keywords:** gut microbiota, LAB, child, Lactobacillus spp., alpha diversity

## Abstract

Acute lymphoblastic leukemia (ALL) treatment, involving chemotherapy, radiotherapy, and pharmacotherapy (antibiotics, antineoplastics) perturbs the gut microbiota in pediatric patients, with enduring effects post-treatment. ALL treatments diminish microbial richness and diversity, favoring pathogenic bacteria. Probiotics may offer promise in mitigating these disruptions and associated side effects. This mini-review explores the impact of ALL treatment on the gut microbiota and the potential benefits of probiotics in pediatric oncology. Probiotics have shown promise in restoring gut microbial balance, reducing treatment-associated side effects, and potentially improving quality of life. However, potential adverse effects, particularly in immunocompromised patients, warrant caution. Notably, there's emerging interest in probiotics’ role in bone health and mineral bioaccessibility. Further research is needed to elucidate probiotics’ mechanisms and their broader impact on pediatric health. Integration of probiotics into ALL treatment and post-treatment regimens offers significant potential for improving patient outcomes and reducing treatment-related complications and long-lasting disruptions, although careful monitoring is essential.

## Introduction

Acute lymphoblastic leukemia (ALL) is the malignant transformation and proliferation of lymphoid progenitor cells. The disease results from the clonic expansion of abnormal lymphoid B lymphocyte progenitors (BCP-ALL) in 80% of all cases, or T lymphocytes (T-ALL) in 20% of all cases, which attack bone marrow, peripheral blood, and extramedullary sites (1, 2). About 75% of paediatric ALL develops from B-cell lineage precursors, with the remainder comprising malignant T-cell precursors (3). ALL used to be intractable, but now has a survival rate of up to 80%–90% (4). The incidence of ALL is highest in children aged 1–4 years and represents 80% of the most common cancer before the age of 15 years (5–7). The treatment of ALL typically involves chemotherapy, radiotherapy, and immunotherapy (8). While these therapies target cancer cells, they often also affect healthy cells and tissues, leading to significant disruptions in the body. One major impact is the destruction of the gut's microbiology, which weakens the immune system. Additionally, these treatments can cause a range of side effects, including hair loss, fatigue, anemia, and weight changes—particularly with chemotherapy (9). Digestive problems and skin issues such as dryness, redness, or itching are common with radiotherapy (10), while immunotherapy may result in fever and high blood pressure (11). Probiotics, as live microorganisms, could significantly contribute to the recovery of the intestinal microflora and mitigate treatment-related side effects (12).

### Acute effects of ALL treatment on gut microbota composition

The human gastrointestinal tract harbors a diverse and ever-changing community of microorganisms, predominantly bacteria (of a number CFU exceeding 10^14^) that exert a substantial influence on the host. Approximately 90% of the this bacterial population is comprised of two phyla: gram-positive Bacillota synonym Firmicutes (including *Bacillus* spp., *Lactobacillus* spp., and *Clostridium* spp.) and gram-negative Bacteroidetes (13). A harmonious equilibrium among bacterial populations is essential for maintaining the homeostatic conditions of the gastrointestinal tract. Traditional treatments for ALL encompass chemotherapy, radiotherapy, and antimicrobial agents, that significantly disrupt this homeostasis (14). Research on gut microbiome perturbation during childhood treatment ALL has identified chemotherapy, antibiotics, immunosuppression, dietary modifications, and direct toxic effects as key contributors to changes in the gut ecosystem (15, 16). Furthermore, the consistently observed deficiency of short-chain fatty acid-producing bacterial taxa in children with ALL may exacerbate these gut ecosystem changes, leading to dysregulated immune responses and potentially increasing the risk of progression from preleukemic clones to overt leukemia (17, 18). Numerous investigations have delved into the ramifications of systemic cancer therapies on intestinal microflora composition (19, 20). Notably, multi-day regimens of high-dose chemotherapy have been associated with a marked reduction in microbial richness and gastrointestinal diversity (21). Furthermore, a discernible shift from gram-positive to gram-negative bacteria is statistically evident (22). Specifically, there is an escalation in the abundance of Bacteroidetes (including Bacteroides and Prevotella) and Proteobacteria (such as Enterobacteria, e.g., *Escherichia coli*, and *Shigella* sp.), alongside an increase in Firmicutes (comprising *Lactobacillus* sp. and *Enterococcus* sp.), while Actinobacteria (including *Bifidobacterium* sp.) exhibit a decrease (23). It was found that the total amount of flora in the stools of children with ALL decreased within 1 week by almost 30% after chemotherapy compared with healthy children (24). In our previous research, we confirmed the decline in bacterial diversity and richness in pediatric hematopoietic stem cell transplantation (HSCT) patients post-cancer treatment. Beneficial bacteria decreased notably from day 0 to day 90 after HSCT. Additionally, we reported a shift in gut microbiome composition, with a dominance of unclassified *Enterococcus* species and Enterococcaceae family during the 3 month period post-HSCT (25). Furthermore, the overuse of antibiotics in ALL treatment promotes the colonization of *Clostridium difficile* (26), an opportunistic pathogen responsible for antibiotic-associated diarrhea, while also reducing the presence of protective species like *Bifidobacterium* spp. (27). Noteworthy, *Clostridium difficile* infections, in conjunction with bloodstream infections, pose grave complications in pediatric oncology and bone marrow transplant recipients (28). A recent study demonstrated that IL-22, derived from the intestinal microbiota, regulates mucosal glycosylation, promotes the growth of the symbiotic bacterium *Phascolarctobacterium*, and enables it to compete with *Clostridium difficile* for succinate, thereby preventing *Clostridium difficile* infection (29).

### Long effects of ALL treatment on gut microbiota composition

Although it is well known that cancer treatment can temporarily alter the composition of normal human microflora, long-term effects have also been demonstrated (30). Data from child and adolescent cancer patients confirmed that intestinal microbial dysfunction persists for months after intensive cancer (31). The microbiological profiles of both patient and sibling control groups are dominated by members of *Bacteroides*, *Prevotella*, and *Faecalibacterium* (31). At the level of the genus, both groups share many common taxa, but the diversity of micro-organisms in the patient group is significantly lower than in the control group. In the control group, higher proportions of *Anaerostipes, Coprococcus, Roseburia,* and *Ruminococcus* taxis were observed in pores with oncological patients (32). Further analysis of different bacterial species between ALL and control groups found that the relative abundance of *Edwardsiella tarda* and *Prevotella maculosa* was reduced in pediatric ALL patients and was positively correlated with interleukin-10 (IL-10) levels (18). It was reported that certain probiotic strains regulated T helper 17 (Th17) cell polarization, which in turn triggered innate lymphocytes to produce interleukin-22 (IL-22). IL-22, a key immune defense cytokine, played a crucial role in maintaining intestinal homeostasis and promoting tissue healing and regeneration (33). In terms of time to oncological treatment during the first 6 weeks of treatment, specifically in the induction phase, the rates of Streptococcaceae and Enterococcaceae were increasing (31). During the consolidation (9 months) and maintenance phases (2–3 years), the frequency of Lachnospiraceae and Clostridiceae decreases and Bifidobacteriaceae, Streptococcacea, and Enterococcaceae appear (34). Thomas et al. (35) found that the microbial composition at the end of therapy differed significantly from that of healthy children. Compared to healthy controls, the microbial diversity of adult patients who survived pediatric ALL and discontinued treatment for at least 5 years before observation was significantly reduced, with exceptional enrichment by actinobacteria. Therefore, microbial dysregulation due to chemotherapy and antibiotics during treatment may have long-term effects on the development of diseases other than obesity or diabetes in adults who have received ALL as children (36).

### Probiotic benefits for post-therapy complications

Probiotic functions that are beneficial promote intestinal restoration and maintenance of homeostasis (37). Probiotics are living microorganisms that confer benefits to the host when administered in suitable dosages (38, 39). At present, probiotic bacteria can be found in a wide range of products, including foods such as yogurt, kefir, and fermented cabbage; dietary supplements, and medicines (40). In human nutrition, probiotic microorganisms are predominantly classified into the following genera: *Lactobacillus, Bifidobacterium, Lactococcus, Streptococcus,* and *Enterococcus* (41). Probiotics have demonstrated numerous beneficial effects on clinical outcomes, including treatment of mucositis, respiratory infections, and antibiotic-related digestion (42, 43). It was discovered that peritonitis patients exhibited a restoration of commensal intestinal bacteria, both in quantity and functionality, which had potentially been eradicated after the treatment (44). Treatment-related diarrhea is one of the most frequent and problematic adverse reactions associated with chemotherapy or radiotherapy in people with cancer. Its reported incidence was up to 50%–80% (45). Intestinal mucositis causing severe treatment-related diarrhea can lead to fluid and electrolyte losses and nutritional deficiencies and could adversely affect quality of life (46, 47). Probiotics may be effective in the prevention or treatment of diarrhea caused by chemotherapy or radiotherapy (48). Potentially, probiotic-mediated microbiota modulation could reduce chemotherapy-associated adverse effects and eradicate multidrug-resistant strains in child patients with hematologic malignancies (49). The beneficial effects of probiotics including a decrease in treatment-associated side effects and nausea and vomiting (49–51) are presented in Table 1. Despite limited research on gut microbiota restoration in patients in remission, our recent study confirmed the combined effect of probiotics and physical exercise on the structure of the intestinal microbiota in pediatric children with ALL in remission (52). The principal findings indicate that exercise and probiotics have a substantial positive effect on microbial alpha diversity.

**Table 1:** Therapeutic effect of probiotics in pediatric oncology patients with acute lymphoblastic leukemia (ALL).

Study	Probiotic	Therapy	Study group	Stage of treatment
Ekert et al. (49)	*Lactobacilli spp.*	No information about dose and time	68 children with leukemia and solid tumors	No data avaible
Wada et al. (50)	*Bifidobacterium breve*	109 freeze-dried, living BBG-01, 8 weeks	42 patients with malignancies	During chemotherapy
Reyna-Figueroa et al. (51)	*Lactobacillus rhamnosus*	5 × 109 CFU twice daily, 1 week	60 children with acute leukemia	Remission induction (after chemotherapy)
Bielik et al. (52)	*Lactobacillus casei*	20 billion CFU lactobacillus casei CNCMI-1518, 8 weeks daily	16 children with acute leukemia	1–3 years after cancer treatment

### The role of probiotics on quality of life

There are significant associations between intestinal microbes and psychoneurological symptoms (PNS), including fatigue, exercise capacity, anxiety, and depression, according to preclinical studies (53). Intestinal microbial dysfunction has been implicated in the development of anxiety and mood disorders in patients. Probiotics can modify the performance of functional brain activity and affect emotional attention, according to additional clinical studies (54). Probiotics have been linked to notable weight gain in underweight pediatric oncology patients exhibiting reduced appetite. By improving gut health, probiotics may reduce symptoms like bloating, diarrhea, and abdominal pain, which can significantly enhance overall well-being and quality of life (55, 56). Furthermore, alterations in mood and sleep quality were detected. Particularly, the research demonstrates progressive enhancements in diverse dimensions of the affective profile, including melancholy, ire, and lethargy, among individuals undergoing probiotic treatment in childhood leukemia (57). Reportedly, the quality of sleep has been enhanced despite the implementation of probiotics in both cancer patients and survivors (58).

### Awareness and adverse effects

Although probiotics have been shown to have considerable advantages, their safety and effectiveness in immunocompromised patients are still unknown; therefore, care should be taken when administering them. It is crucial to take into account potential adverse effects associated with probiotics, such as systemic infections, gastrointestinal disturbances, skin reactions, acquisition of antibiotic resistance genes, detrimental impacts of probiotic metabolites, and atypical immune stimulation (54). Although probiotics are generally regarded as safe for healthy individuals, there is limited evidence to suggest otherwise for certain populations, including oncology patients in pediatrics. Pediatric patients can develop systemic infections, detrimental metabolic processes, heightened immune stimulation in vulnerable individuals, and the transfer of resistance genes (59). An extensive epidemiological study on patients revealed that instances of infectious endocarditis and bacteremia caused by Bifidobacteria and Lactobacillus were exceedingly rare, accounting for a mere 0.05%–0.4% of the total cases (60). Infections that are linked to probiotic strains of lactobacilli are exceedingly uncommon. Land et al. (61) and Avcin et al. (62) described two patients who were administered probiotic lactobacilli and bifidobacterium and subsequently developed bacteremia and sepsis. The risk of infection was found to be correlated not only with individual factors but also with the specific type and dosage of probiotics. Five out of 1,530 patients were found to have probiotic-related bacteremia, according to one study; however, probiotic therapy did reduce the frequency and severity of diarrhea in these cancer patients (63).

## Future perspectives

**Figure 1:**
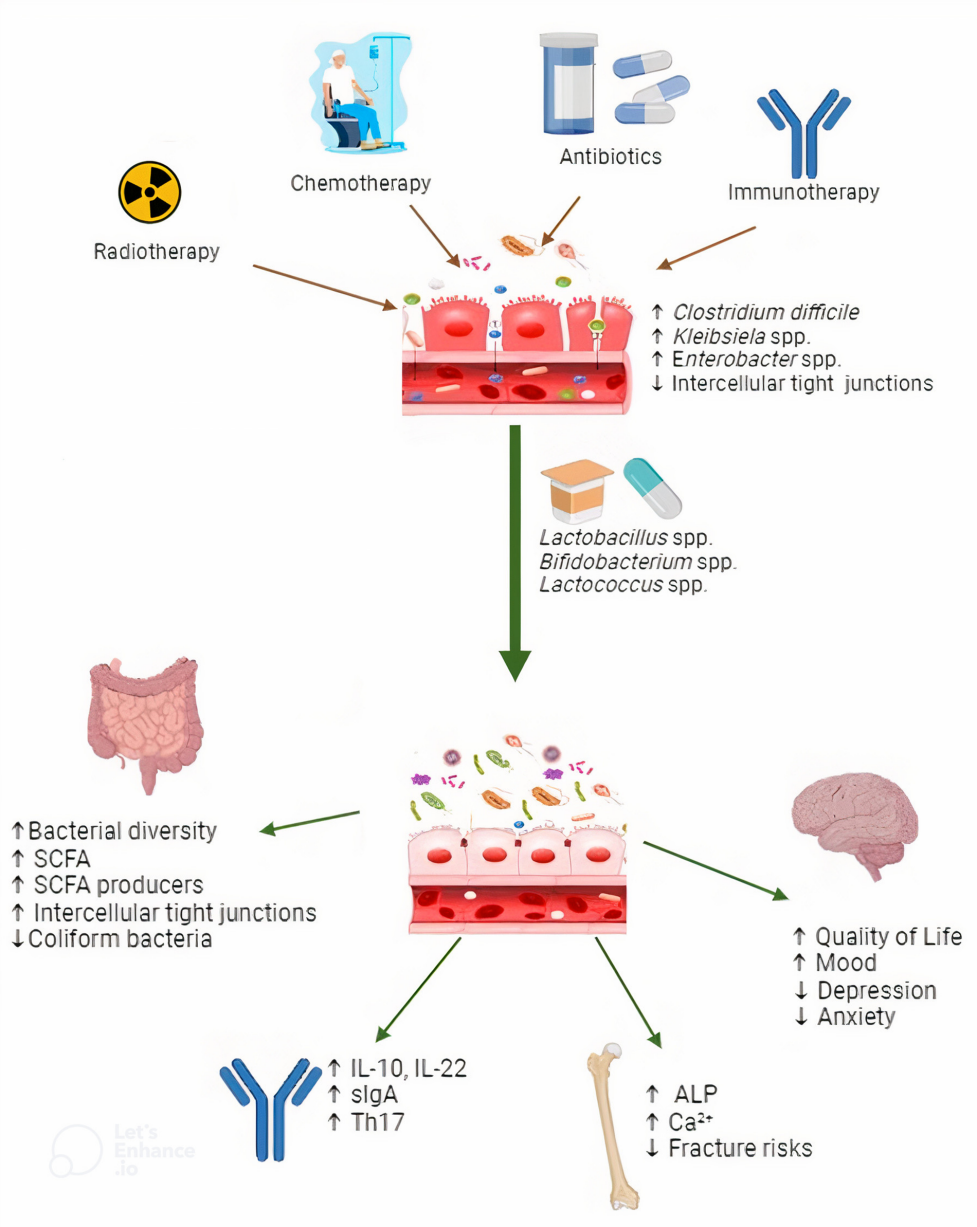
The effects of antibiotics, chemotherapy, radiotherapy, and immunotherapy on the gut microbiota in pediatric oncology patients with acute lymphoblastic leukemia (ALL), and the role of probiotics in mitigating these effects. Probiotics help restore microbial balance by promoting the growth of beneficial bacteria that produce short-chain fatty acids (SCFAs), inhibiting the growth of harmful coliform bacteria, and maintaining intestinal barrier integrity, including the restoration of intercellular tight junctions. They influence immune modulation, reflected in increased levels of IL-10, IL-22, secretory IgA (sIgA), and Th17 cells, which are markers of enhanced immune responses. Additionally, probiotics contribute to bone health by supporting calcium absorption and promoting bone remodeling, as indicated by elevated alkaline phosphatase (ALP) levels, ultimately lowering the risk of fractures. Probiotics also positively impact mental health by reducing symptoms of anxiety and depression, thereby improving the overall quality of life in these patients.

The majority of probiotic research in recent years has focused on their effect on the composition of gut microbiota. However, there is still a fascinating and relatively unexplored area regarding how these microorganisms function through specific molecules, metabolic pathways, and transport carriers within their host, pediatric oncology patients (Figure 1). As mentioned earlier, reduced bone mineral density, a known side effect, is present during and persists a couple of years after the ALL treatment (64–66). Chemotherapy disrupts the balance between bone resorption and formation by affecting osteoclasts, leading to increased bone resorption (67). In children with ALL, ALP levels serve as a marker for monitoring bone health during treatment. Emerging research suggests that probiotics may influence bone metabolism and, as a result, potentially reflect these ALP levels (68). Therefore, it is critical to investigate factors such as mineral bioaccessibility and bioavailability associated with probiotic use, as well as shifts in the gut microbiota composition (69). Future strategies to improve intestinal permeability in pediatric ALL patients may focus on reducing the consequences of compromised gut barrier function, such as elevated zonulin levels (70). Interventions like targeted probiotics and dietary modifications could potentially mitigate intestinal inflammation and restore barrier integrity, thereby addressing the underlying factors that lead to increased zonulin and disrupted gut permeability. We think this area warrants further investigation and could yield valuable insights into the broader effects of probiotics on pediatric bone health.

## Conclusion

Despite limited research, this mini-review outlines the intricate relationship between acute lymphoblastic leukemia (ALL) treatment, gut microbiota, and the potential benefits of probiotics. It highlights the severe impact of chemotherapy and antibiotics on gut microbial diversity, leading to long-lasting changes even after treatment. Probiotics emerged as a promising solution, offering restoration of beneficial bacteria, reduction of treatment-related side effects, and potential improvements in quality of life for ALL patients. However, we advise caution regarding the risk of adverse reactions, especially in individuals with compromised immune systems. Overall, the integration of probiotics into ALL treatment regimens holds significant potential for enhancing patient outcomes and mitigating treatment-related complications. Based on current scientific evidence, we contend that, with cautious administration, the pros of probiotics for pediatric oncology patients outweigh the potential cons.
